# Supplementary material for: Xeroderma Pigmentosum Type C Primary Skin Fibroblasts Overexpress HGF and Promote Squamous Cell Carcinoma Invasion in the Absence of Genotoxic Stress
Source: Cancers (Basel). 2024 Sep 26;16(19):3277. doi: 10.3390/cancers16193277 (PMC11475422; doi:10.3390/cancers16193277)
Supplement: Supplementary file 1 [file cancers-16-03277-s001.zip › cancers-2987966-supplementary.pdf]

Supplementary Figures

A

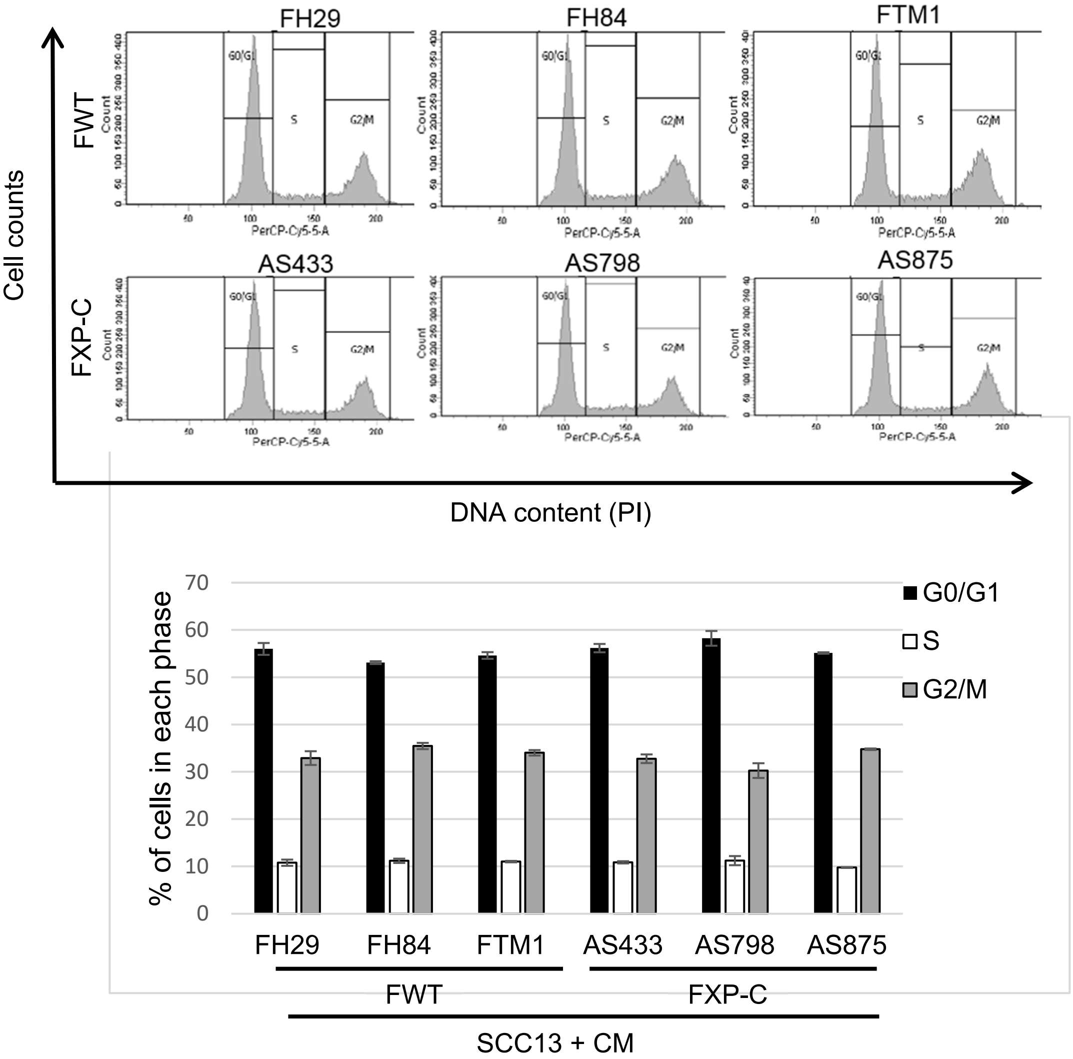

B

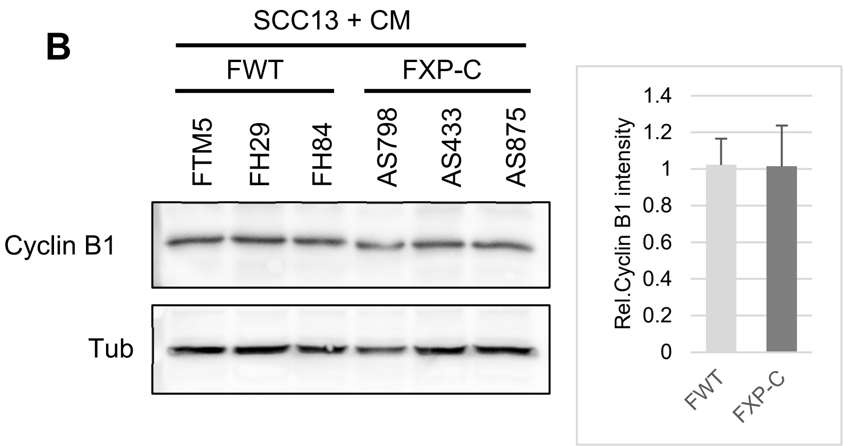

Figure S1. Impact of XP-C CM on SCC13 cell cycle. Upper panel: flow cytometric profile of SCC13 cell cycle after co-culturing with WT (FWT) or XP-C (FXP-C) fibroblast CM. Lower panel shows that there were no detectable significant differences in the cell cycle phases of SCC13 cells incubated either WT or XP-C fibroblast CM. Results are the mean ± SD of three independent experiments. (B) Western blot analysis of cyclin B1 in lysates of SCC13 cells cultured in either WT or XP-C fibroblast CM. Beta tubulin labelling was used as loading/transfer control. Right panel: quantification of cyclin B1 expression in the different conditions. Values are presented as means ± SD.

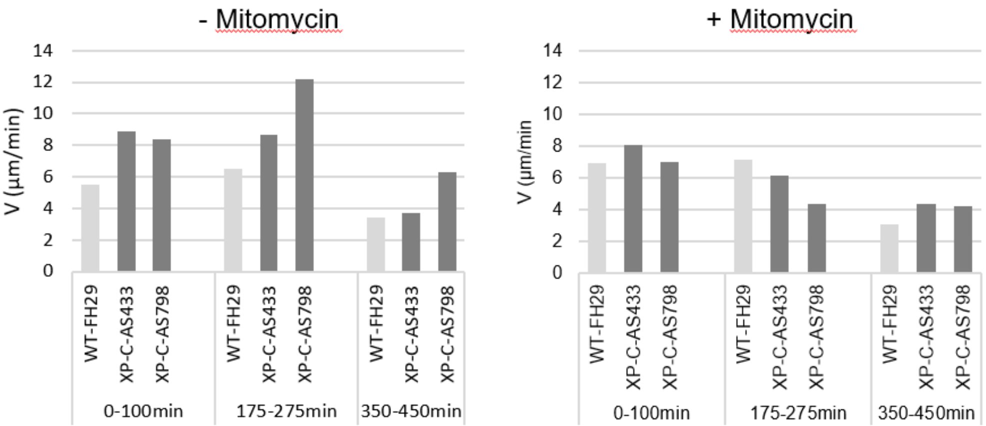

Figure S2. Effect of mitomycin C on scratch closure kinetics of SCC13 cells cultured in CM from either WT or XP-C fibroblasts. Histogram of scratch wound closure of SCC13 cancer cells in either the presence or the absence of CM from either WT or XP-C primary fibroblasts as indicated.

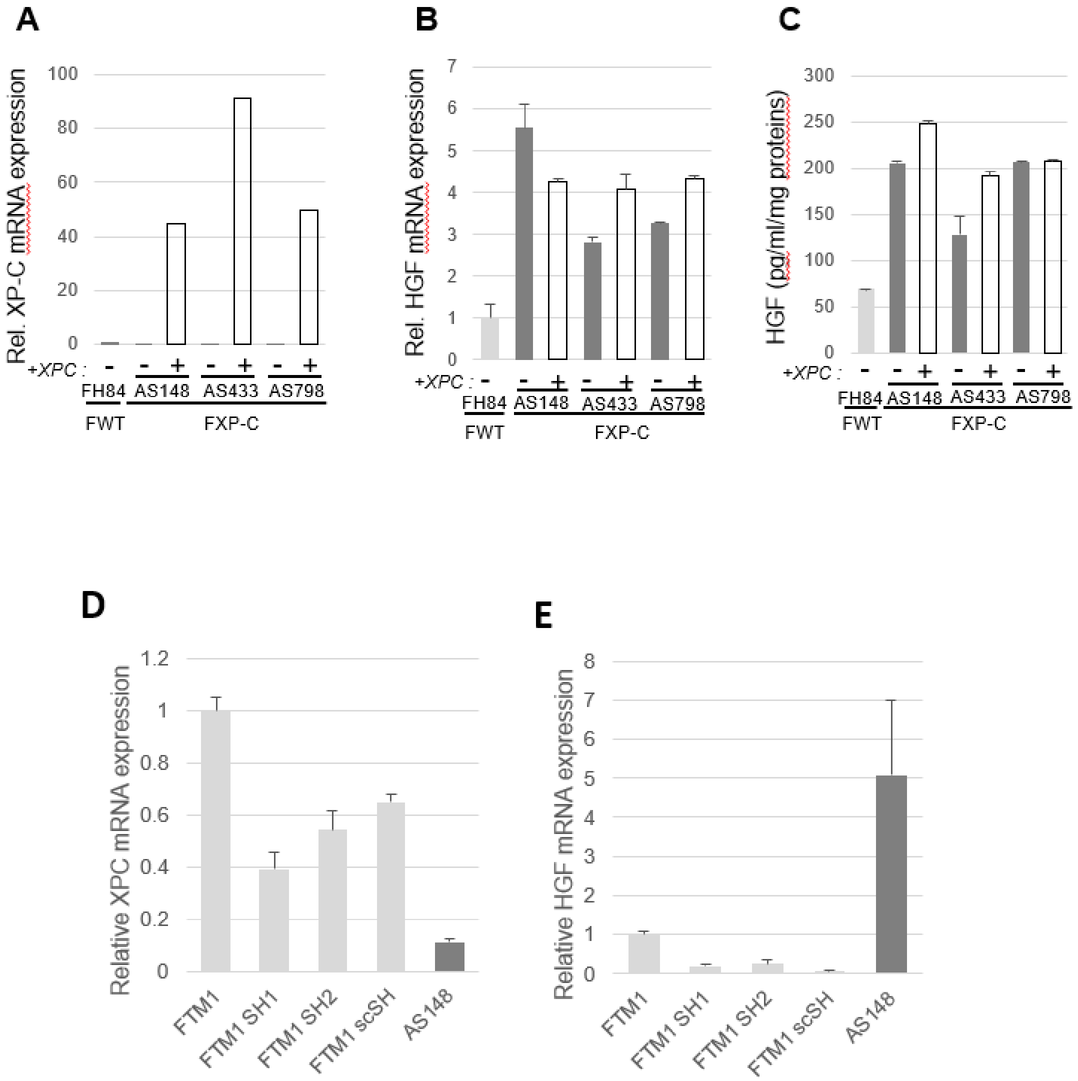

Figure S3. HGF expression in *XPC* shut down WT fibroblasts and in reverted XP-C fibroblasts. **A,** Average XPC mRNA levels in WT or XP-C fibroblasts stably complemented for *XPC expression* (-: non reverted; +: after reversion and selection). **B,** Relative mRNA expression of *HGF/SF* in 3 independent primary fibroblast cell strains either before (-) or after (+) genetic complementation. **C,** Relative secretion of *HGF/SF* in 3 independent primary fibroblast cell strains either before (-) or after (+) genetic complementation**. D,** Relative mRNA expression of *XPC* mRNA in primary fibroblast cell strains presenting attenuated expression of *XPC* after *XPC-SH* mRNA transduction; note that *HFF/SF* is substantially reduced in XC-C SH transduced fibroblasts. **E,** Relative HGF/SF mRNA expression in three WT primary cell strains compared to the WT FTM1 cell strains. Relative *HGF* mRNA (over)expression in XP-C (AS 148) fibroblasts is shown as control.

Supplementary Figure S4

pSTAT3
86 kDa

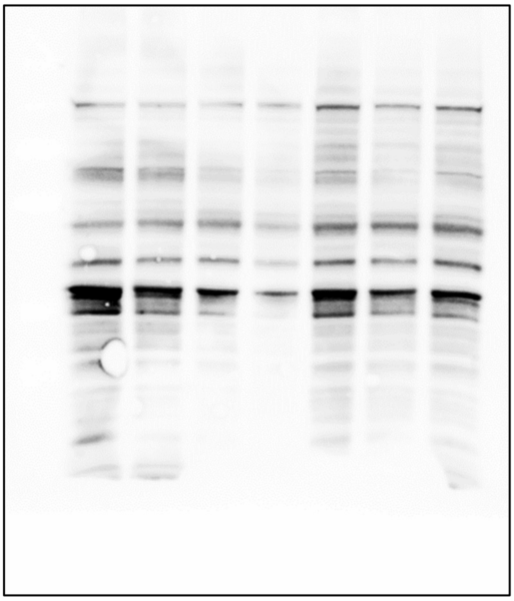

STAT3
86 kDa

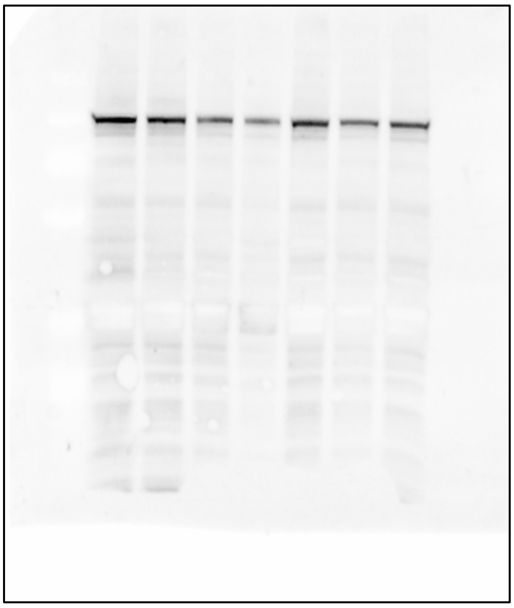

Tubulin
50 kDa

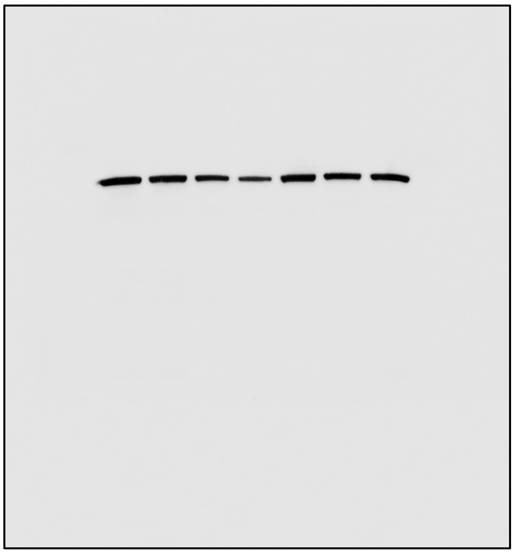

Figure 3 D

pJNK 1, 2
55 kDa

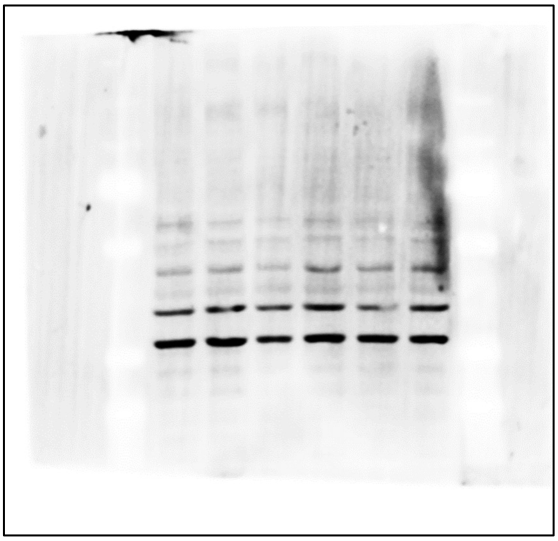

JNK 1, 2
55 kDa

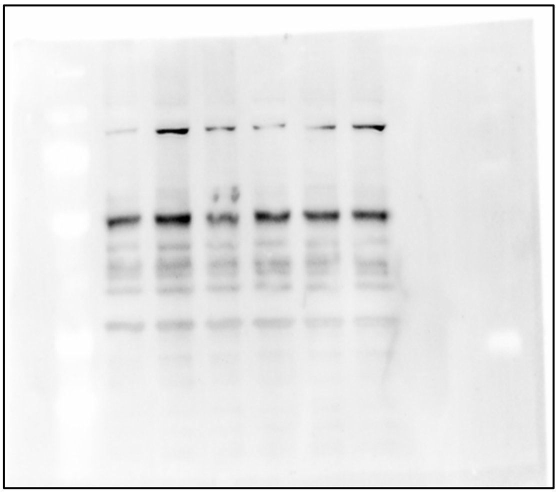

Tubulin
50 kDa

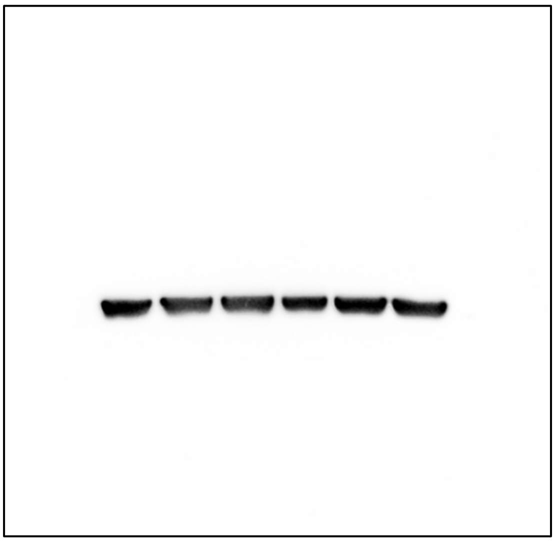

Figure 3 E

pERK
42 kDa

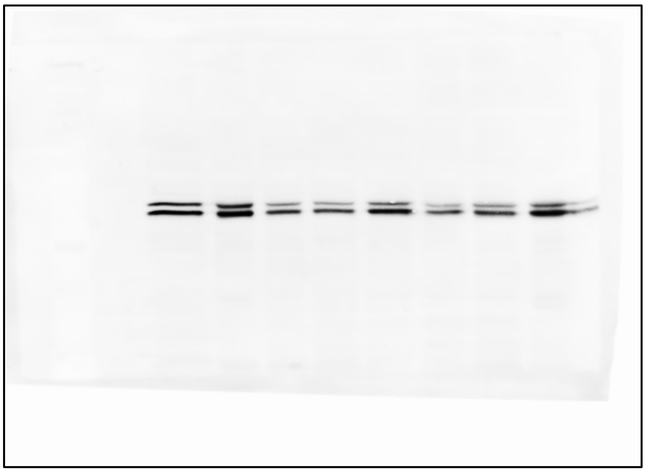

Tubulin
50 kDa

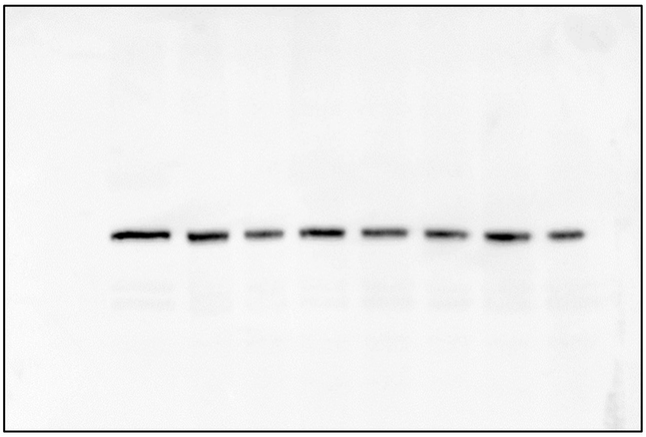

ERK
42 kDa

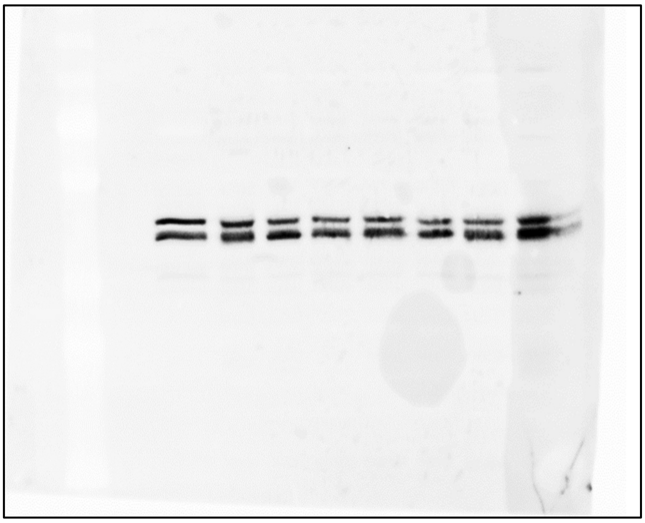

Figure 3 F

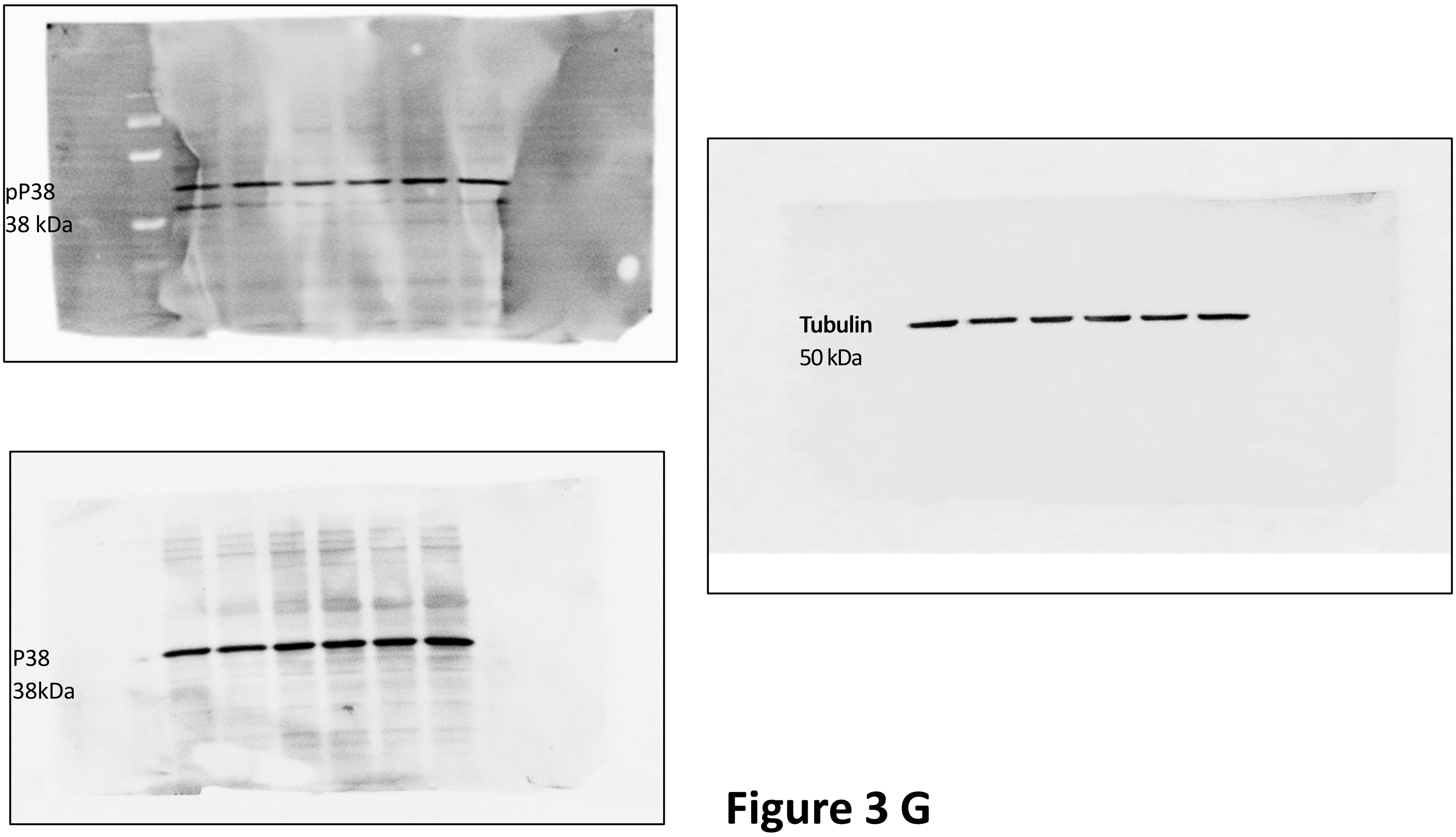

Figure 3 G

pSTAT3
86 kDa

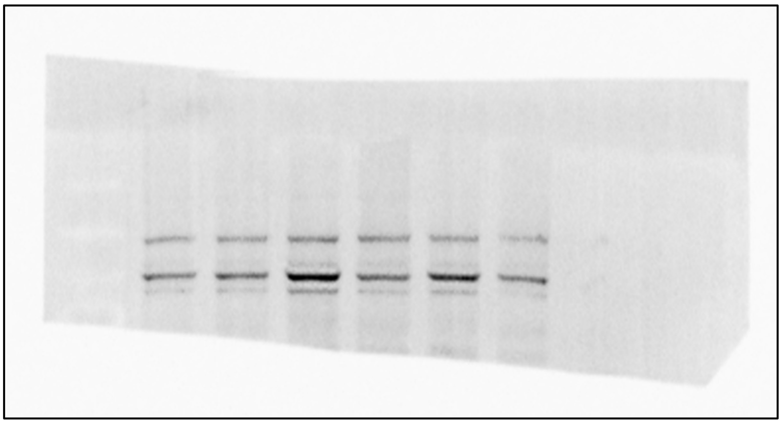

STAT3
86 kDa

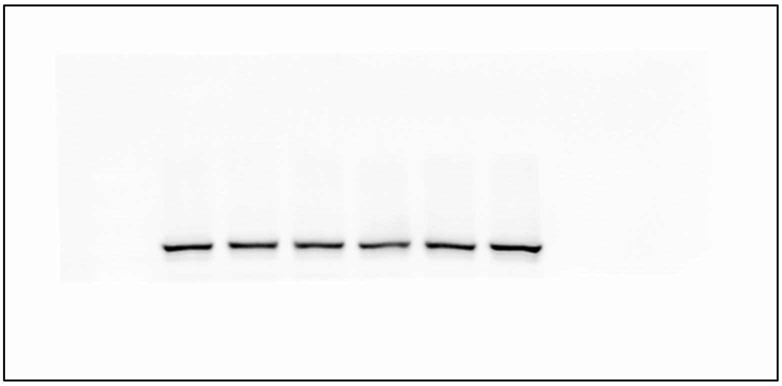

Tubulin
50 kDa

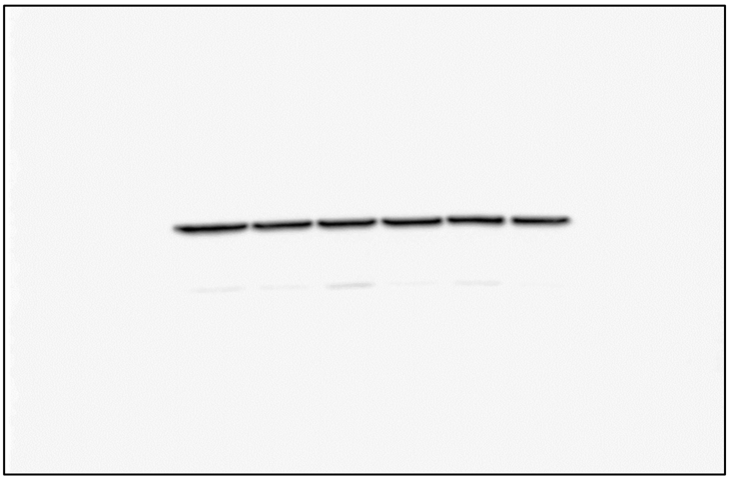

Figure 4 B

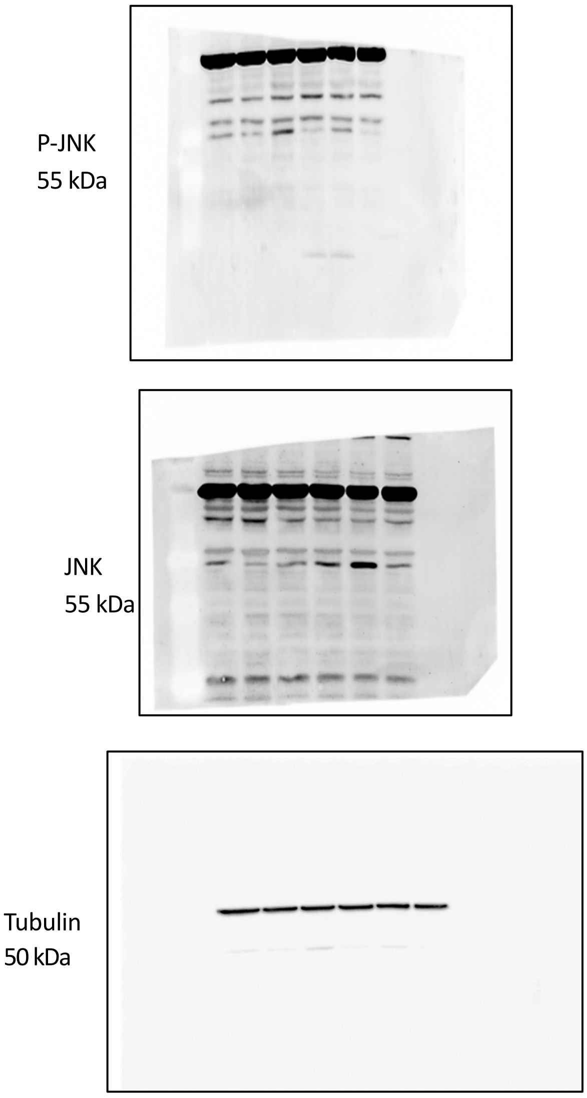

Figure 4 B

Supplementary Figure S4: uncropped images of western blots show in Figure 4 & 5

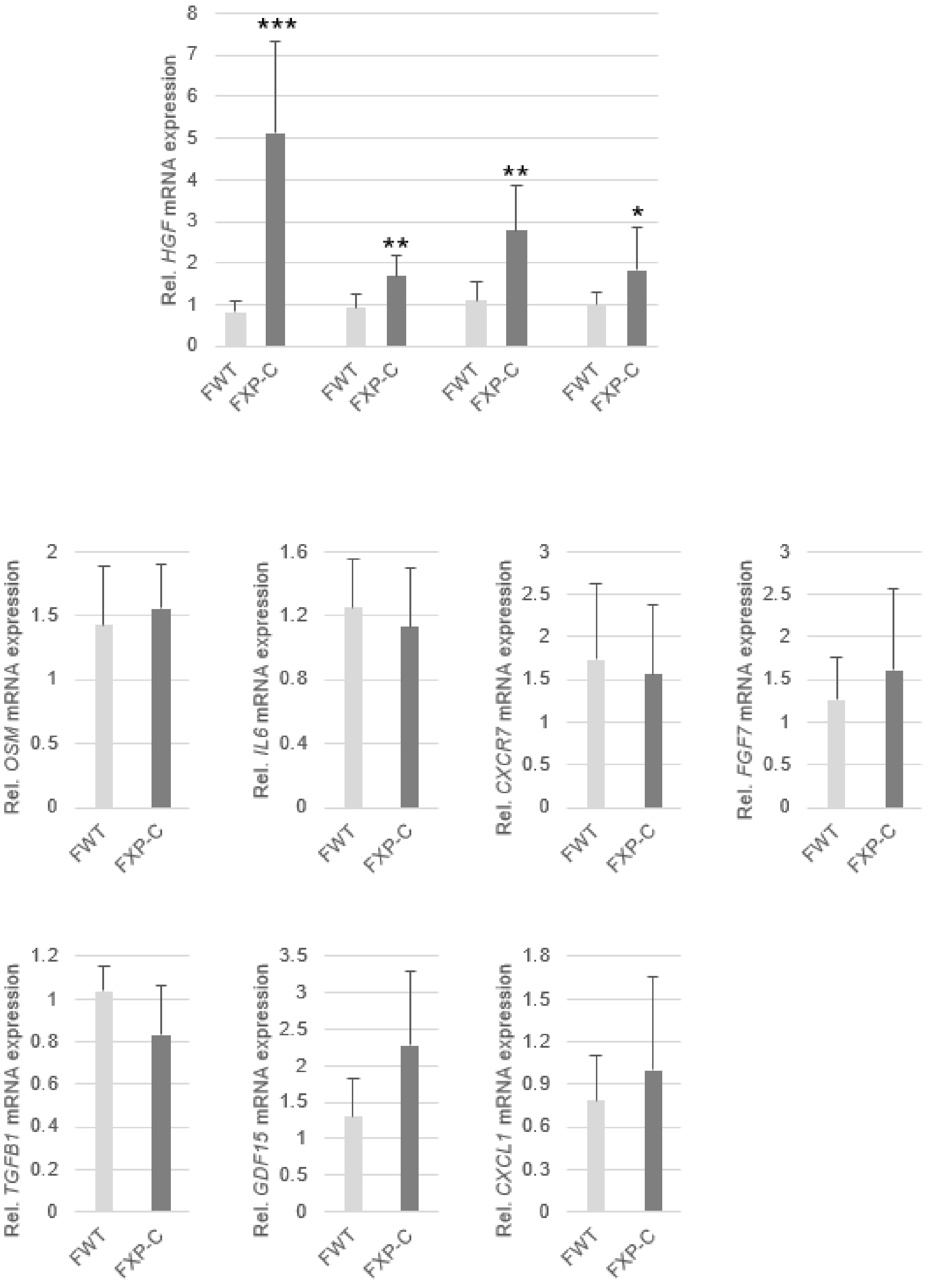

Figure S5: Relative mRNA levels of HGF, OSM, IL6, CXCR7 and FGF7 in WT compared to XP-C fibroblsasts without any treatment. Values are presented as means ± SD of at least two independent experiments. *P < 0.05, **P < 0.01, ***P < 0.001. Note that only HGF mRNA was significantly incrased solely in XP-C fibroblasts.

Table S1. Patients and cell characteristics. .

Fibroblasts	Ethnic origin	Phenotype	XP-C mutation	Age at biopsy	Clinical characteristics	Onset of tumors
FTM1	Caucasian	NA	NA	40 years	NA	NA
FH29	Caucasian	NA	NA	30 years	NA	NA
FH84	Black	NA	NA	28 years	NA	NA
FMD	Caucasian	NA	NA	16 years	NA	NA
FTM5	Caucasian	NA	NA	25 years	NA	NA
AS728		NA	NA		NA	NA
AS148	Caucasian	XP-C	c.1643_1644delTG; p.Val548Alafsx25	12 years	Multiple BCCs and SCCs, face and exposed areas	9 years
AS433	Caucasian	XP-C	c.1643_1644delTG; p.Val548Alafsx25	2 months	NA	NA
AS798	Caucasian	XP-C	c.1643_1644delTG; p.Val548Alafsx25	2 years	Photoaging (hands), ephelides, actinic keratosis	NA
AS202		XP-C	c.1643_1644delTG; p.Val548Alafsx25	Fetus	NA	NA
AS875		XP-C		20 years		
AS629		XP-C	c.1643_1644delTG; p.Val548Alafsx25		NA	NA
AS673		XP-C	c.1643_1644delTG; p.Val548Alafsx25		NA	NA

Table S2. Primer sequences and pre-designed Taqman assays used for quantitative real-time PCR.

Gene Name	Forward primer (5′ to 3′)	Reverse primer (5′ to 3′)
HGF	TCACGACATGACATGACTCC	AGCTTACTTGCATCTGGTTCC
XPC	CTGCGCAGCCAGAAATCC	TGTCTTCATTCATGGTAGCC
CD24	AACTAATGCCACCACCAAGG	CCTGTTTTTCCTTGCCACAT
TGFB1	GCGTGCTAATGGTGGAAAC	CGGTGACATCAAAAGATAACCAC
CXCL12/SDF1	GTGCCCTTCAGATTGTAGC	GTCTTTGCCCTTTCATCTC
IL6	GGCACTGGCAGAAAACAACC	GCAAGTCTCCTAATTGAATCC
EGF	TACCGTTAAGATACAGTGTAGGCAC	ATCACAACTCATTTTGGCAAAATC
LIF1	CAGCATCACTGAATCACAGAGC	ATCACAACTCATTTTGGCAAAATC
36B4	TGCTCAGTACCCCATTCTA	AGTTATGAAACATCCCCACAGGG
SB34	GCATCAGTACCCCATTCTATCAT	AGGTGTAATTCCGTCTCCACAGA
GAPDH	TGACAAGGTGCGGGCTCCCTAGG	CCAAGGCTGTGGGCAAGGTCAT

Gene Name	Taqman Gene Expression Assay Reference
FGF7	Hs00940253_m1
CXCL12	Hs00171022_m1
PPIA	Hs99999904_m1
GAPDH	hS99999905_m1
RPL01	Hs99999902_m1
